# Single-access, non-contrast transcatheter aortic valve implantation, the ultimate minimalist approach: a case report

**DOI:** 10.1093/ehjcr/ytae040

**Published:** 2024-01-25

**Authors:** Mario E Diaz Nuila, Ashish Gupta, Mohammad Alkhalil

**Affiliations:** Cardiothoracic Centre, Freeman Hospital, Freeman Road, Newcastle-upon-Tyne NE7 7DN, UK; Cardiothoracic Centre, Freeman Hospital, Freeman Road, Newcastle-upon-Tyne NE7 7DN, UK; Cardiothoracic Centre, Freeman Hospital, Freeman Road, Newcastle-upon-Tyne NE7 7DN, UK; Translational and Clinical Research Institute, Newcastle University, Framlington Place, Newcastle-upon-Tyne NE2 4HH, UK

**Keywords:** Aortic stenosis, Transcatheter aortic valve implantation, Renal impairment, Single access, Case report

## Abstract

**Background:**

Transcatheter aortic valve implantation (TAVI) is an established treatment for patients with symptomatic severe aortic stenosis. Patients with previous renal transplant are considered as a high-risk cohort who may develop procedural complications related to vascular access and renal impairment post-TAVI.

**Case summary:**

Herein, we report a case of an 88-year-old male who presented with progressive dyspnoea. His transthoracic echocardiogram revealed severe aortic stenosis with a peak gradient of 75 mmHg and impaired left ventricle systolic function (an estimated ejection fraction of 40%). He had a background of kidney transplant with progressive decline in renal function, requiring the formation of left arm arteriovenous fistula in preparation for future dialysis. He was successfully treated with TAVI using a single vascular access site without administering contrast media.

**Discussion:**

Single-access, non-contrast TAVI is feasible when treating renal transplant patients with severe aortic stenosis and limited vascular access. The current minimalistic approach should be used only in highly selective patient cases.

**Learning points**

- Zero-contrast transcatheter aortic valve implantation (TAVI) is feasible only in highly selective patient cases.
- Single-access TAVI is a safe approach and is an option in patients with challenging vascular access.

## Introduction

Aortic stenosis is a common pathology in patients undergoing renal replacement therapy and has a faster rate of progression when compared with the general population.^1^ Renal transplant, however, is not associated with a worsening of the severity of an established aortic stenosis.^2^ Nonetheless, patients with renal transplant have high mortality rates when requiring valve replacement, which may approach 20% per year.^3^

Transcatheter aortic valve implantation (TAVI) becomes an established treatment for patients with severe aortic stenosis.^4^ Transcatheter aortic valve implantation in patients with renal transplant may pose procedural challenges related to limited vascular access and renal impairment post-TAVI.

## Summary figure

**Figure:**
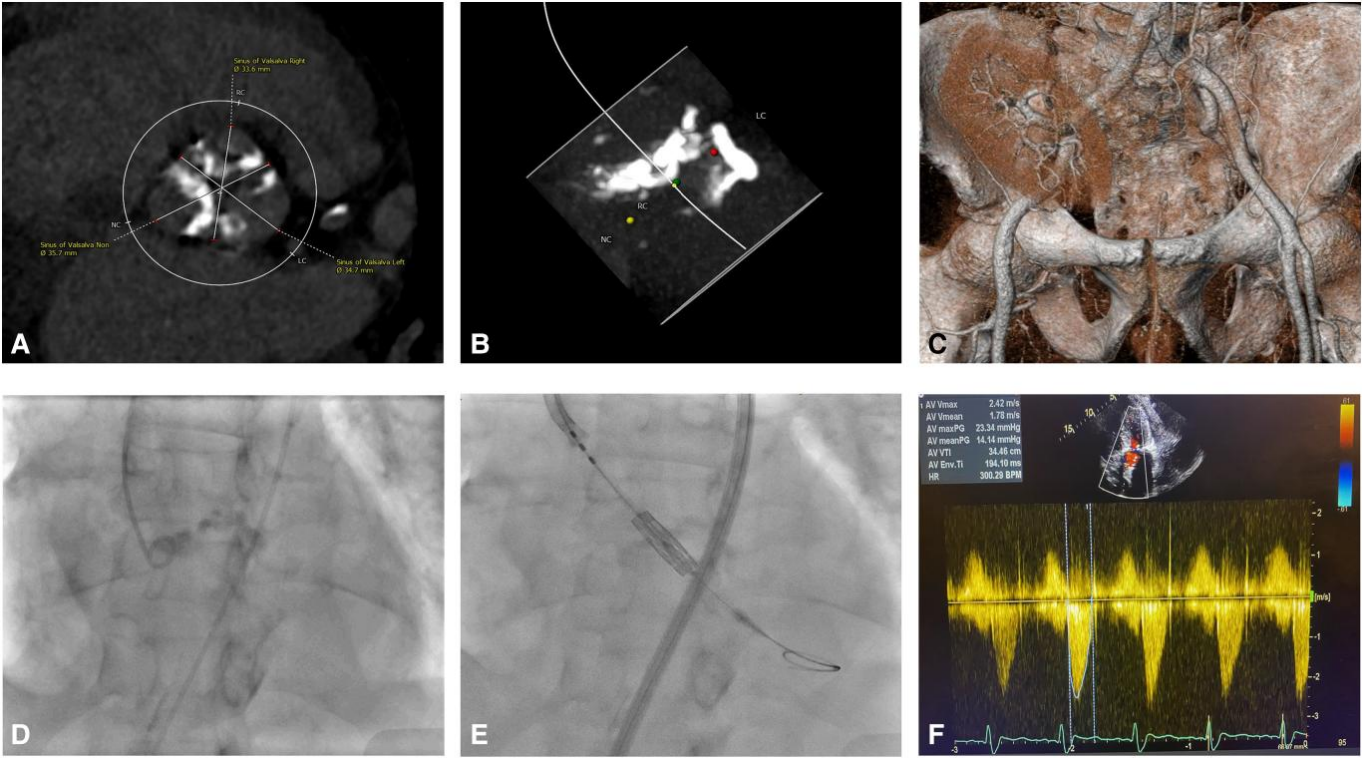
Procedural planning using single-access transcatheter aortic valve implantation without the need for contrast media. Calcium distribution of the aortic valve leaflets, aortic annulus, and left ventricle outflow tract on computed tomography (*A* and *B*). The presence of the vascular pedicle of the transplanted kidney from the right iliac artery excluding this site as a potential access for the transcatheter heart valve (*C*). The presence of angiographic calcium distribution–aided precise positioning of the transcatheter heart valve (*D* and *E*). Post-implant echocardiogram illustrating optimal valve function (*F*).

## Case presentation

**Figure 1:**
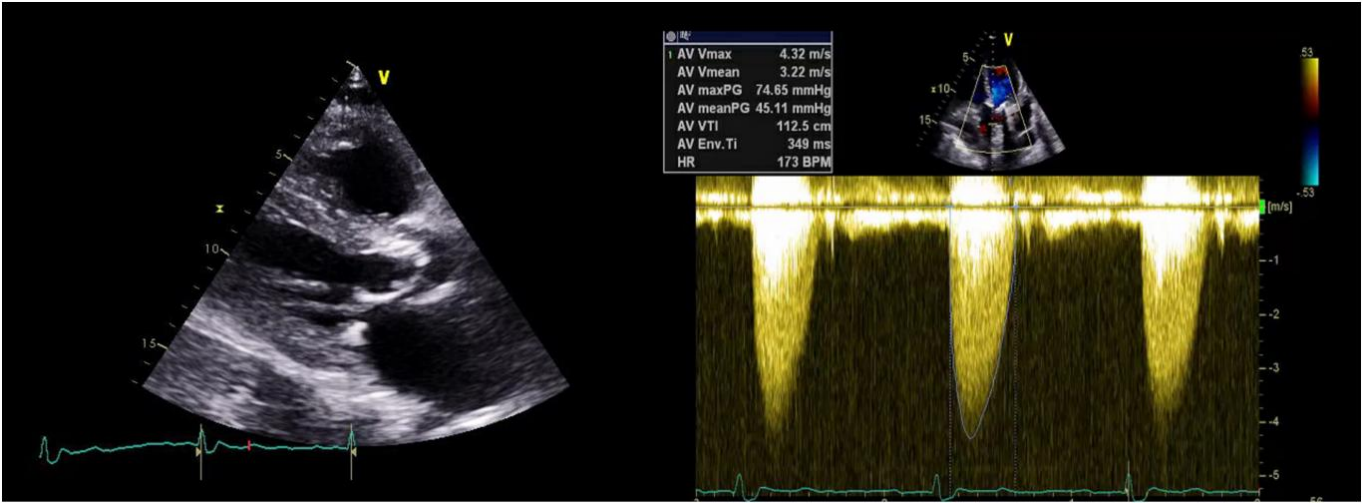
A long-axis echocardiographic image and continuous wave Doppler demonstrating severe aortic stenosis.

**Figure 2:**
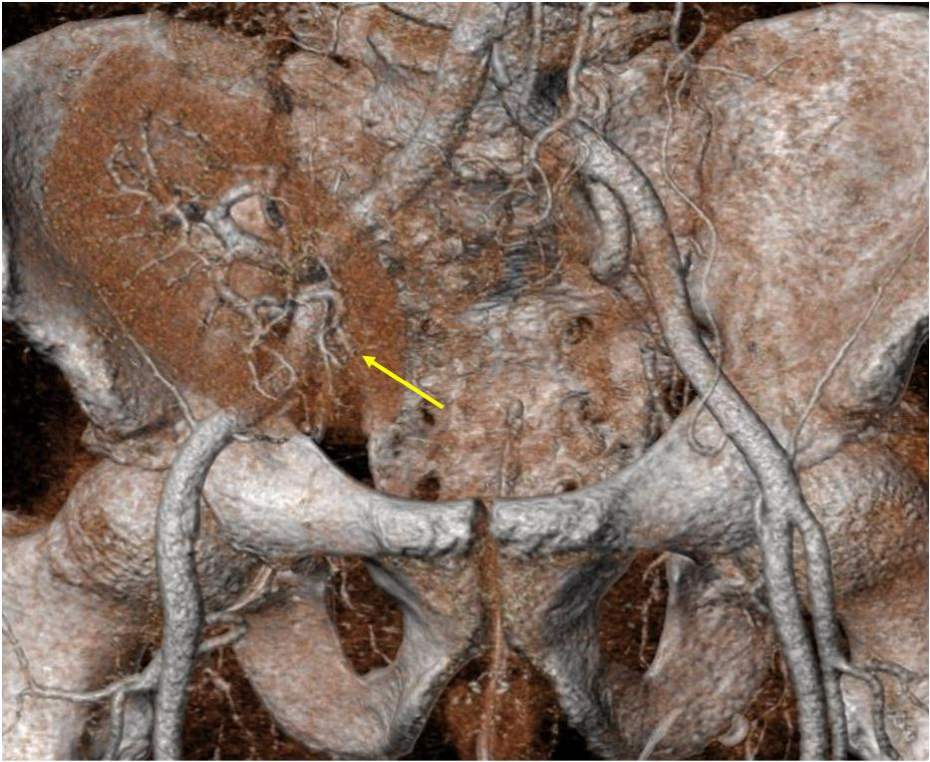
A computed tomography image demonstrating a heterotopic kidney transplant with a vascular pedicle coming off the right iliac artery (arrow) and a suitable left femoral approach for a transcatheter heart valve implantation.

An 88-year-old man was admitted with progressive dyspnoea and fluid overload. There was no history of chest pain or syncope. He had a background of immunoglobulin A (IGA) nephropathy and underwent renal transplant in 2011. His renal function was gradually deteriorating with an estimated glomerular filtration rate of 13 mL/min/1.73 m^2^. Prior to his admission, left arm arteriovenous fistula was performed in preparation for future dialysis. His transthoracic echocardiogram revealed severe aortic stenosis (a peak gradient of 75 mmHg, a mean gradient of 45 mmHg, and an aortic valve area of 0.88 cm^2^) and global impairment of left ventricle systolic function with an estimated ejection fraction of 40% (*Figure 1*). He underwent ultra-low-dose contrast computed tomography (CT) in preparation for TAVI with no decline in his renal function. The CT confirmed heavily calcified aortic valve leaflets and a suitable transfemoral approach for TAVI. His heterotopic transplant kidney was supplied from the right iliac artery, making this site less favourable for vascular access (*Figure 2*). Given the absence of chest pain, regional wall motion abnormalities on echocardiography, or calcified obstructive proximal coronary arteries segments on CT, coronary angiography was not planned to avoid the use of contrast media.

**Figure 3:**
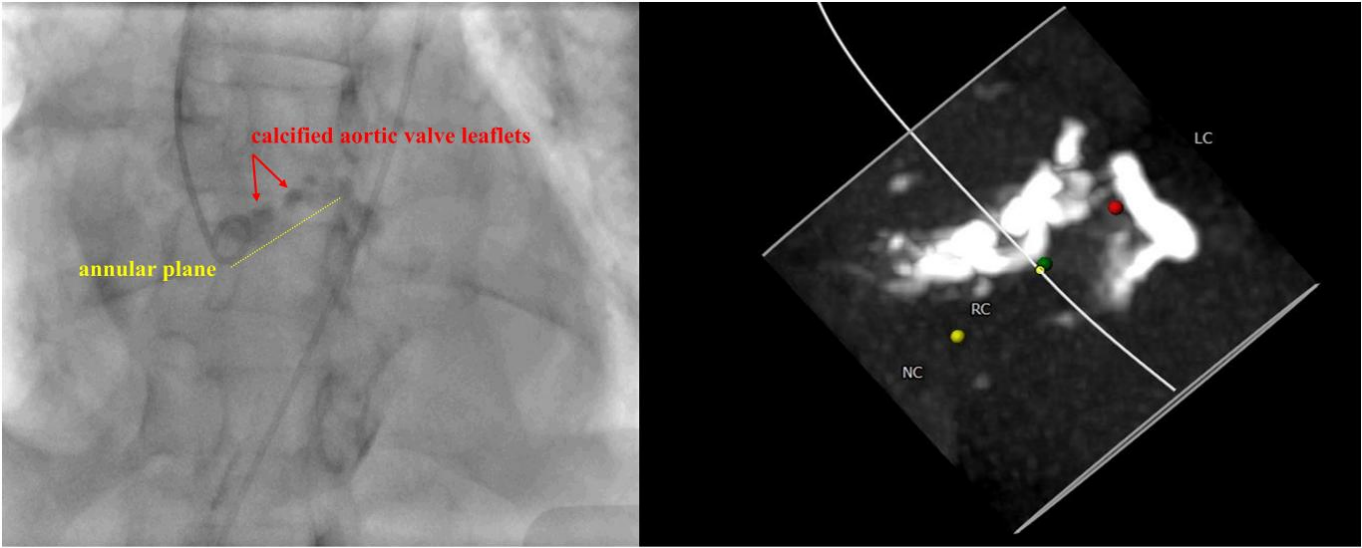
Left panel, a pig-tail catheter position in the non-coronary cusp to identify the annular plane (dotted line) and the distance to the calcified aortic valve leaflets (arrows). The right panel highlights the comparable calcified landmarks between fluoroscopy and computed tomography.

**Figure 4:**
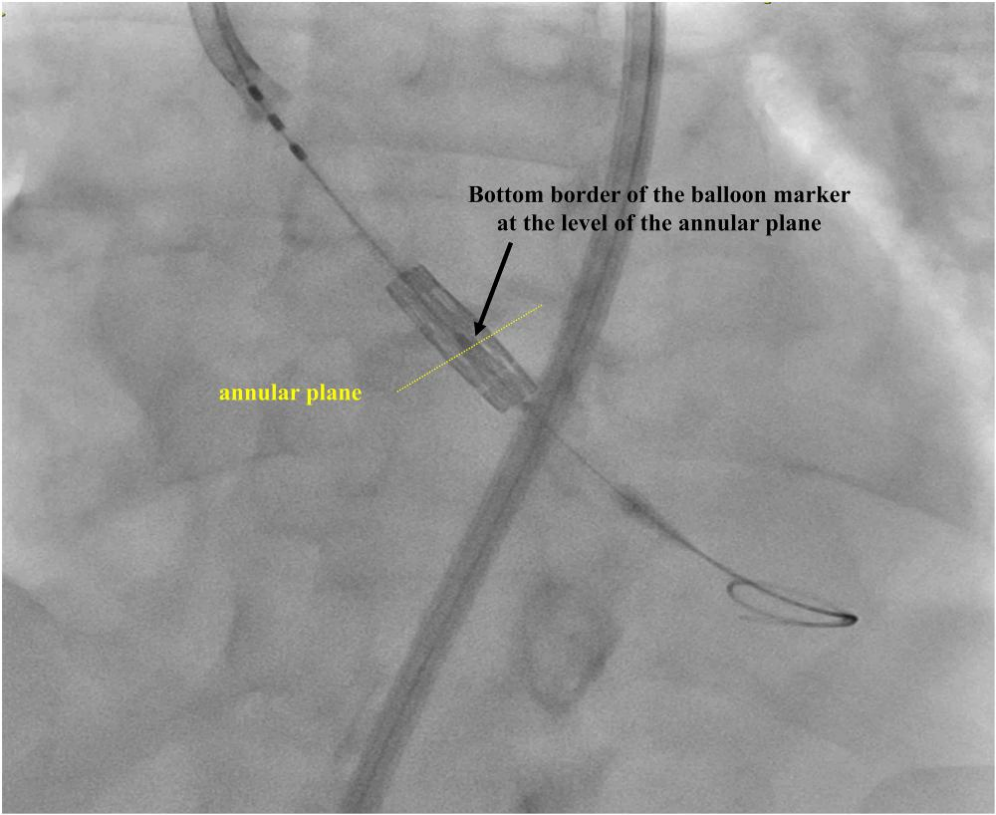
Positioning the middle balloon marker of the transcatheter heart valve below the calcified aortic valve leaflets with the bottom border of the marker at the level of the annulus (dotted line).

The procedure was planned using the left common femoral artery (single vascular access as an unpalpable right radial artery, functional fistula of the left arm, and vascular pedicle of the transplanted kidney from the right iliac artery), aiming to avoid using contrast to preserve the failing transplanted kidney. An ipsilateral second vascular access was planned (but not obtained) as a bail-out vascular strategy. Access was obtained using ultrasound and a 6-French sheath was inserted in the left femoral artery, and subsequently upsized to a 14 Fr eSheath (no second vascular puncture was performed). A 6-French pig-tail catheter was positioned in the non-coronary cusp under fluoroscopy. An acquisition was obtained to identify the landmarks of calcified leaflets and annulus (*Figure 3*; see Supplementary material online, *Video S1*). Calcium distribution on fluoroscopy was comparable with the identified calcium on CT, which helped in guiding an optimal positioning of the transcatheter heart valve (*Figure 3*). Subsequently, the pig-tail catheter was withdrawn, and the stenotic aortic valve was crossed using an amplatz catheter in a standard fashion and exchanged for a pre-shaped left ventricle wire. Central venous access was not obtained, and pacing was tested for stability using sterile alligator clips attached to the patient (via fine gauge needle) and the left ventricle wire (positioned in the apex). The middle balloon marker of 26 mm Sapien Ultra (Edwards Lifesciences) was fully positioned below the calcified aortic valve leaflets with the bottom border of the marker at the level of the annulus (*Figure 4*). This was guided using the calcium silhouette visible at the bottom border of the pig-tail catheter. The transcatheter heart valve was deployed under rapid pacing via the left ventricle wire (see Supplementary material online, *Video S2*). The total radiation time was 7 min and 59 s.

**Figure 5:**
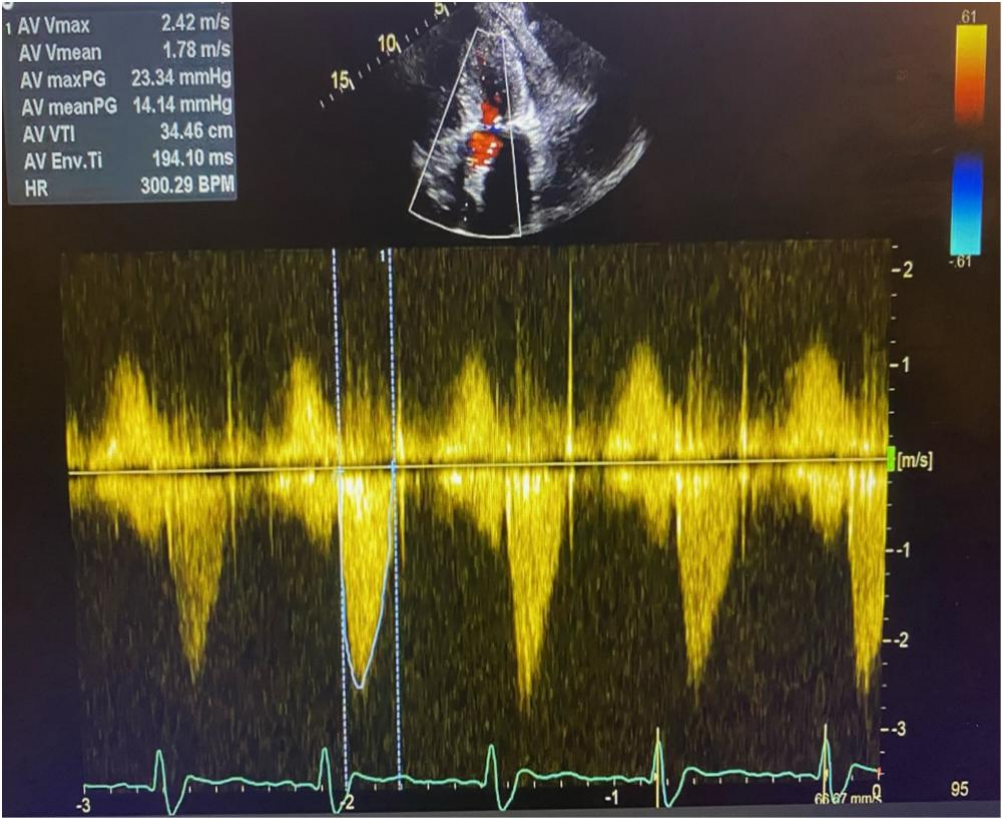
An echocardiogram post-procedure demonstrating optimal valve function.

Following deployment, invasive haemodynamic assessment did not reveal any gradient across the valve. Echocardiography and invasive measures revealed no evidence of aortic regurgitation. No conduction system disturbances were noted. Vascular access was closed in a standard fashion without complications. The patient underwent a post-procedural echocardiogram, which demonstrated an improvement in the left ventricle ejection fraction of up to a rate of 50% with a mean gradient of 14 mmHg (*Figure 5*). There was no meaningful difference in renal function post-TAVI. The patient was symptomatically well at a 4-week follow-up.

## Discussion

This case demonstrates that adequate pre-procedural preparations allowed successful TAVI implantation in a highly challenging patient. A good understanding of the anatomy enabled us to deliver precise implantation without the need for a second vascular access or the use of contrast media in this patient with limited vascular access who was at a high risk of permanent dialysis.

Patients with end-stage renal disease have an increased rate of aortic valvular calcifications that is approximately three times faster than non-dialysis patients.^1,5^ Renal transplant may reduce hospitalization rates for patients with aortic stenosis compared with those with maintained dialysis^1^; however, their in-hospital mortality rate was 14% following surgical valve replacement.^3^ Transcatheter aortic valve implantation is an established alternative to surgical aortic valve replacement (AVR) in patients with prohibitive or high surgical risk.^4^ Data comparing clinical outcomes between TAVI and surgical aortic valve replacement in patients with advanced renal disease are limited.^6^ Mir *et al*.^6^ reported lower mortality rates with TAVI at 30 days but a comparable rate of renal failure and 1-year death when compared with surgery.

The effect of TAVI on renal function is complex. Acute kidney injury following TAVI has been reported in up to 50% of cases.^7^ Certain procedural factors are recognized to be associated with worsening renal function during TAVI. This includes exposure to contrast media, athero-embolic phenomenon, hypotension, and bleeding events. In our patient, there was a significant risk of permanent dialysis given the decline in the function of the transplant kidney. Therefore, we elected to avoid the use of contrast during the TAVI procedure and to rely on non-contrast anatomical landmarks that are present during fluoroscopy. Additionally, routine coronary angiography was not performed to minimize the exposure to contrast media prior to the TAVI procedure. The patient did not report exertional chest pain and there was a lack of regional wall motion abnormalities on echocardiography or severe obstructive calcifications of the proximal coronary artery segments on CT. This selective approach was demonstrated to be safe in patients undergoing TAVI.^8^ Moreover, data on routine coronary revascularization are not supported by the recent ACTIVATION randomized clinical trial.^9,10^

Vascular access was another challenge in this case. The presence of the vascular pedicle of the transplanted kidney from the right iliac artery would exclude the right common femoral artery as a potential access site. The presence of the large bore sheath or any interaction between guidewires and the vascular pedicle may compromise blood flow to the transplanted kidney. Therefore, the left femoral approach was deemed a safer option. The use of the non-femoral approach such as axillary or carotid artery would carry an additional risk of vascular complication, bleeding, and hypotensive episodes that may worsen kidney function and lead to permanent dialysis.^11^ We elected not to concomitantly use a pig-tail catheter during valve deployment given the functioning fistula of the left arm and the non-palpable right radial artery. More importantly, single-access TAVI (without the use of a pig-tail catheter) has been reported to be a safe strategy. It was associated with a shorter procedural time and lower use of contrast media.^12^ Moreover, single-access TAVI has a comparable procedural success to dual access with similar rates of major vascular complications.^12^ Whilst the right brachial artery could have been used, this may add additional vascular risk, and the fluoroscopic anatomical features provided sufficient landmarks to safely deploy the transcatheter heart valve without the need to use a pig-tail catheter. The fluoroscopic calcium distribution within the aortic valve leaflets, the aortic annulus, and the left ventricle outflow tract was comparable with that visible on CT and allowed a precise implantation of the transcatheter heart valve. Post-implantation, there was no echocardiographic evidence to suggest aortic regurgitation, and invasive measurements revealed an excellent functioning valve.

In our patient, a bail-out strategy for vascular complication was planned via an ipsilateral puncture of the superficial femoral artery, if needed. In the possible scenario of our failing to achieve haemostasis using a vascular closure device, a safety peripheral balloon would be delivered via the same vascular access site for the transcatheter heart valve. The plan was to maintain access to the left femoral artery and to ensure that the guidewire was not withdrawn before achieving adequate haemostasis. This would allow adequate time to safely obtain a distal access in the superficial femoral artery to percutaneously manage vascular complication or to prepare the operating theatre for surgical repair.

In conclusion, our case demonstrated the feasibility of implanting a transcatheter heart valve using a single-access strategy and obviated the need for the use of contrast media in a patient at a high risk of permanent dialysis. The current minimalistic approach requires good procedural planning and an understanding of the anatomical landmarks and should be used only in highly selective patient cases.

## Supplementary Material

ytae040_Supplementary_Data

## Data Availability

Data presented in this manuscript are available from the corresponding author on reasonable request.

## Lead author biography

Dr Mohammed Alkhalil is an interventional cardiologist and structural heart specialist at the Freeman Hospital, Newcastle-upon-Tyne, UK. He underwent clinical training at the Royal Victoria Hospital in Belfast, obtained his PhD (DPhil) at the University of Oxford, and secured his clinical fellowship at the Toronto General Hospital in Canada.

## Supplementary material

**Consent:** In accordance with COPE guidelines, patient-informed consent was obtained.

**Funding:** None declared.
