# Correction to: Single-access, non-contrast transcatheter aortic valve implantation, the ultimate minimalist approach: a case report

**DOI:** 10.1093/ehjcr/ytae276

**Published:** 2024-06-04

This is a correction to: Mario E Diaz Nuila, Ashish Gupta, Mohammad Alkhalil, Single-access, non-contrast transcatheter aortic valve implantation, the ultimate minimalist approach: a case report, *European Heart Journal - Case Reports*, Volume 8, Issue 2, February 2024, ytae040, https://doi.org/10.1093/ehjcr/ytae040

In the originally published version of this article, there was a typographical error in author Mohammad Alkhalil’s name.

This has been corrected.

